# A Bioinformatic Analysis Predicts That Cannabidiol Could Function as a Potential Inhibitor of the MAPK Pathway in Colorectal Cancer

**DOI:** 10.3390/cimb46080506

**Published:** 2024-08-05

**Authors:** Julianne du Plessis, Aurelie Deroubaix, Aadilah Omar, Clement Penny

**Affiliations:** 1 Department of Internal Medicine, Oncology Division, Faculty of Health Sciences, University of the Witwatersrand, 7 York Road, Parktown, Johannesburg 2193, South Africa; 2489917@students.wits.ac.za (J.d.P.); aurelie.deroubaix@wits.ac.za (A.D.); aadilah.omar@wits.ac.za (A.O.); 2 Life Sciences Imaging Facility, Faculty of Health Sciences, University of the Witwatersrand, 7 York Road, Parktown, Johannesburg 2193, South Africa

**Keywords:** cannabidiol, MAPK, EGFR, Ras/Raf, colorectal cancer

## Abstract

Colorectal cancer (CRC), found in the intestinal tract, is initiated and progresses through various mechanisms, including the dysregulation of signaling pathways. Several signaling pathways, such as EGFR and MAPK, involved in cell proliferation, migration, and apoptosis, are often dysregulated in CRC. Although cannabidiol (CBD) has previously induced apoptosis and cell cycle arrest in vitro in CRC cell lines, its effects on signaling pathways have not yet been determined. An in silico analysis was used here to assess partner proteins that can bind to CBD, and docking simulations were used to predict precisely where CBD would bind to these selected proteins. A survey of the current literature was used to hypothesize the effect of CBD binding on such proteins. The results predict that CBD could interact with EGFR, RAS/RAF isoforms, MEK1/2, and ERK1/2. The predicted CBD-induced inhibition might be due to CBD binding to the ATP binding site of the target proteins. This prevents the required phosphoryl transfer to activate substrate proteins and/or CBD binding to the DFG motif from taking place, thus reducing catalytic activity.

## 1. Introduction

Colorectal cancer (CRC) is the third most diagnosed cancer. It has the second highest mortality rate worldwide, with the five-year survival rate decreasing significantly in the more advanced stages [1,2]. Metastatic CRC, in most cases, remains incurable, but treatment has been improved by cytotoxic chemotherapy and targeted therapy [2]. Cancer cells generally form when gene mutations lead to defects within cellular regulatory systems. This results in cells undergoing continuous, unregulated proliferation and growth and resistance to apoptosis, both with and without growth signals being present [3,4,5]. These characteristics are all features usually controlled through a wide variety of signaling pathways, which can often be dysregulated by being either hyper- or under-activated [4,5,6]. The most commonly dysregulated pathways with or lacking mutations in critical genes or proteins in colorectal cancer are the MAPK, EGFR, and Wnt/β-catenin signaling pathways [7]. These pathways are all involved in various essential cellular processes, such as cell migration, proliferation, angiogenesis, apoptosis, differentiation, and survival, all being implicated in cancer formation and progression [7,8].

The epidermal growth factor (EGF) and EGF receptor (EGFR) play an essential role in mammalian cells by regulating cellular growth, differentiation, proliferation, and survival [9]. EGFR is a member of the receptor tyrosine kinase (RTK) family linked to multiple cancers, especially metastatic colorectal cancer [10]. The activation of the EGFR will lead to the cascade-like activation of various downstream signaling pathways, such as MAPK and PI3K [10,11]. Dysregulated or overexpressed signaling pathways, specifically MAPK, are often present in CRCs, with the inhibition of these pathways being targets for cancer treatment [7,12].

Cannabidiol (CBD) is the primary non-psychoactive compound of Cannabis sativa, a plant commonly used as a medicinal agent to provide relief from pain and anxiety. In contrast, the psychotropic compound is ∆9-tetrahydrocannabinol (THC) [13]. In vitro studies have previously reported the ability of CBD to treat various cancer types [14,15,16]. Specifically in CRC cells, apoptosis was induced following CBD treatment by regulating pro- and anti-apoptotic proteins. Anti-apoptotic protein expression, such as NOXA, was significantly increased in CRCs due to CBD-facilitated stimulation of the production of reactive oxygen species (ROS) [17]. Although CBD has been shown to induce cytotoxic effects, such as apoptosis in CRC [17], little is known about the effect of CBD on other properties of cancer cells. As a prelude to in vitro studies, the effect/s of CBD on the proteins involved in the MAPK pathway (EGFR, RAS, RAF, MEK, and ERK) are predicted here using a bioinformatic- and docking-based study, which could provide information regarding the functioning of these proteins. Similar studies have been conducted to determine if and to which proteins a drug, specifically CBD, will bind to; however, this approach has not yet been used [18,19,20]. Known protein target prediction and molecular docking software and databases were used to identify which amino acid residues are involved in the predicted interactions [15,16].

## 2. Materials and Methods

### 2.1. Chemical Entities of Biological Interest

In the preliminary stages of this in silico analysis, the Chemical Entities of Biological Interest/ChEBI database (https://www.ebi.ac.uk/chebi/) (accessed on 13 May 2021) was used to obtain the 3D structure of CBD. “Cannabidiol” was entered into the database as a search term to generate a list of associated compounds. The 3D structure of CBD, with the correct molecular weight, was downloaded as a spatial data file (.sdf) and used during further analyses, using High-Throughput Docking.

### 2.2. High-Throughput Docking

The 3D structure of CBD was submitted to the HT-Docking website (https://www.cbligand.org/HTDocking/search_struct.php) (accessed on 13 May 2021) to identify proteins that interact with CBD. These were ranked according to their highest-to-lowest docking scores, indicating the strength of the interaction between CBD and a potential target protein. Proteins with a docking score higher than 6.5 were selected for further analysis from this list.

### 2.3. Reactome

The Reactome Pathway Browser (https://reactome.org/PathwayBrowser/) (accessed on 18 May 2021) was used to identify the subcellular location and pathway(s) of the proteins identified during HT Docking. The proteins were filtered based on the main pathway they are involved in and their function, after which proteins involved in the MAPK pathway were selected for further evaluation.

### 2.4. Protein–Ligand Docking Prediction

Proteins directly involved in the MAPK pathway were chosen, due to the large number of MAPK proteins identified, for further docking studies (see Table 1 for the list of proteins). The crystal structure of each of these proteins was downloaded from the Protein Data Bank (https://www.rcsb.org/) as a .pdb file. However, since these structures represent proteins complexed with a ligand, it was necessary to remove these using CCDC GOLD software (2022.3). The new ligand-free protein structure was downloaded as a .pdb file. Subsequently, this file, together with the 3D structure of CBD, was uploaded to the CB Dock 2 website (https://cadd.labshare.cn/cb-dock2/index.php) (accessed 27 June 2022), which simulated the binding of CBD to each protein. The three best docking-solution files were downloaded, each with its relative Vina scores. Vina scores, like docking scores, indicate the strength of the protein–ligand-binding interaction, but whereby the more negative the score, the stronger the interaction between protein and ligand [17]. The Vina score obtained from the predicted CB docking indicates the strength of the interaction between CBD and the specific protein that CBD is docked onto.

### 2.5. Amino Acid Residue Identification at Docking Sites

**Figure 1:**
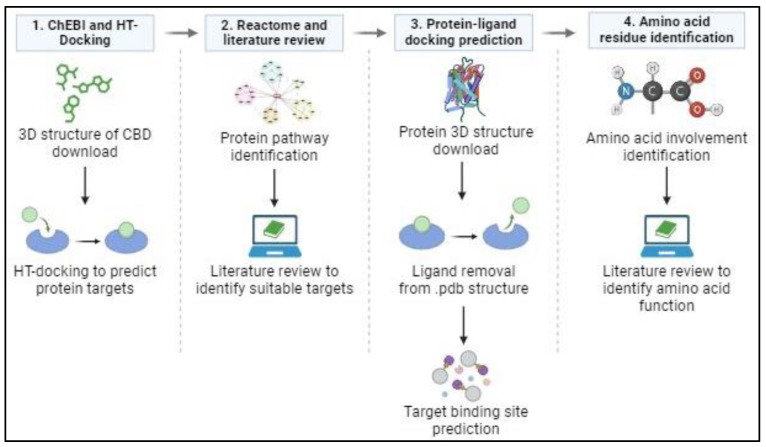
In silico analysis protocol. (**1**) The 3D structure of CBD was obtained from ChEBI as a .sdf file and uploaded to HT-Docking for binding prediction. (**2**) Pathway identification via Reactome and a literature review were conducted to identify suitable proteins involved in CRC. (**3**) Protein crystallography structure, in complex with a ligand, was obtained from Protein Data Bank, and then the ligand was removed using CCDC GOLD software. This new protein structure and CBD structure were uploaded to the CB dock 2 website to perform docking simulations. (**4**) The docked file was downloaded and opened using LigPlot to identify the amino acid residues involved in CBD binding to the protein. A literature review was then performed to predict the effect/s that CBD binding to these specific amino acids and binding sites could have on the target proteins’ functionality.

The CBD–protein complex from the three optimal docking solutions was uploaded to the LigPlot^+^ v.2.2. program obtained from the European Bioinformatic Institute (https://www.ebi.ac.uk/thornton-srv/software/LigPlus/) (accessed on 2 August 2022). This program identified the amino acids involved in the predicted bonds formed between CBD and the docking site of each protein analyzed. The type of bond formed between amino acid residues and atoms from CBD was also identified. From this, a literature review was conducted to determine the possible role these amino acid residues may play in the activity and functioning of the protein and to predict what effect CBD could have on protein activity and, subsequently, the MAPK pathway (Figure 1).

## 3. Results

HT-Docking was used to predict which of the 607 proteins available in this database interacted with CBD and the relative strength of this interaction. Of these, 48 proteins linked to CRC were identified to interact strongly with CBD (Supplementary Table S1). Reactome-based pathway investigation of these proteins indicated that CBD could interact with a wide variety of MAPK and MAPK-like proteins and signified that CBD could play an important role in inhibiting or limiting the function of the MAPK pathway. An additional docking analysis using LigPlot revealed that CBD formed strong hydrogen bonds with various amino acid residues in the selected MAPK-pathway proteins (Table 1). These interactions were stabilized by hydrophobic interactions between the atoms of CBD and amino acids found in or near the binding site (Table 1). The top four scores are represented by BRAF (−8.9), EGFR (−8.8), KRAS (−8.5), and MEK1 (−8.0) (Table 1). Also represented in Table 1 are the amino acid residues involved in forming hydrogen bonds with CBD and those amino acids responsible for hydrophobic interactions with CBD. For this study, EGFR, KRAS, BRAF, and MEK are discussed below.

**Figure 2:**
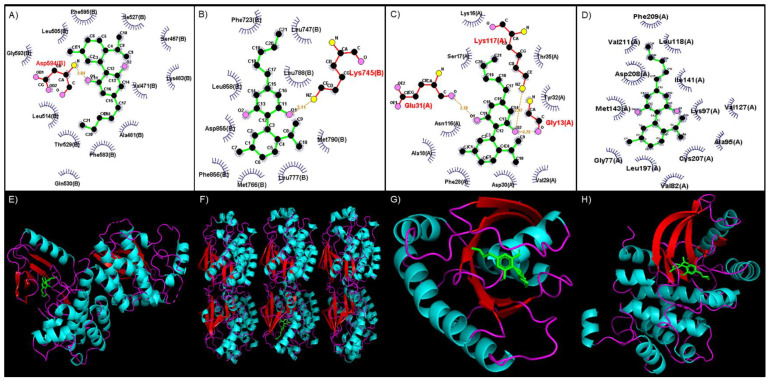
Binding site of CBD to various MAPK pathway proteins visualized with LigPlot. (**A**) BRAF, (**B**) EGFR, (**C**) KRAS, and (**D**) MEK1. Green—CBD; red—hydrogen bond; blue—hydrophobic interaction. The 3D conformation of CBD binding to various MAPK-pathway proteins visualized with PyMol. (**E**) BRAF, (**F**) EGFR, (**G**) KRAS, and (**H**) MEK1. Green—CBD; turquoise—helix; red—sheets.

The binding site of CBD to the kinase domain of BRAF indicates that CBD would form a hydrogen bond with the Asp594 residue, which occurs between the amino group (-NH2) of Asp594 and the alcohol group (-OH) side chain of CBD (Figure 2A). CBD binds to EGFR by forming a hydrogen bond with the Lys745 residue between the -NH2 group of Lys745 and the -OH side chain of CBD (Figure 2B). The binding of CBD to KRAS involves Gly13, Glu31, and Lys117, which form a hydrogen bond with CBD. These interactions occur between the -NH2 group of Gly13, -OH side chain and Lys117, and the oxygen group of Glu31 and the second oxygen atom of CBD (Figure 2C). A MEK1 interaction with CBD occurs mainly through hydrophobic interactions within the binding site, which includes amino acid residues such as Gly77, Val82, Ala95, Lys97, Leu118, Val127, Ile141, Met143, Leu197, Cys207, Asp208, Phe209, and Val211 (Figure 2D).

## 4. Discussion

The results of the docking analysis predicted that CBD would bind to a variety of MAPK signaling-pathway proteins, with various strengths and types of bonds formed between the protein and CBD. These results indicate that CBD could work in a multifaceted mechanism, binding and potentially inhibiting this signaling cascade in several different ways.

### 4.1. CBD Could Act as a Tyrosine Kinase Inhibitor

The in silico analysis of the interaction between CBD and EGFR predicted that CBD would interact with EGFR, a transmembrane receptor involved in the phosphoryl transfer from EGFR to target protein [21]. EGFR is involved in many signaling pathways, of which the aberrant activation leads to the initiation and progression of cancer and CRC [22,23,24,25]. The hydrogen bond between CBD and EGFR involves the Lys745 residue, which is highly conserved across all types of kinases. A single Lys→Arg mutation leads to a complete loss of catalytic activity of EGFR, which highlights the importance of this residue in phosphate transfer and catalysis [26,27]. CBD binding to this residue could diminish the catalytic activity of EGFR due to its importance in the ability of EGFR to function normally. Resistance to first- and second-generation EGFR TKIs arises due to the Thr790→Met mutation [28,29]. The Thr790 residue is located in the bottom of the ATP binding pocket, and the Thr790→Met mutation causes a conformational change, which reduces the ability of first- and second-generation TKIs to bind to the ATP pocket [18,29]. This Thr790→Met mutation also increases the affinity of the mutant receptor of ATP, which reduces the efficacy of first- and second-generation inhibitors [18,27,28,29].

Tyrosine kinase inhibitors (TKIs) are compounds which function by inhibiting the tyrosine kinase residues (TKRs) from catalyzing phosphorylation of the target protein’s tyrosine residue, thereby blocking target protein activation [17,18,30]. These TKIs can be grouped into first- and second-generation TKIs based on the treatment line into which these inhibitors fall. First-generation inhibitors are the “first line” of treatment used to treat a type of cancer and act as inhibitors to a variety of tyrosine kinases [31,32]. Second-generation TKIs are inhibitors used when resistance to first-generation inhibitors has been acquired due to mutations involved in critical sites in the kinases [33]. Since CBD forms a hydrophobic interaction with Met790 (Figure 2B)—the mutated isoform—it could potentially hinder the binding of ATP to the binding site, potentially preventing the phosphorylation of downstream target proteins. This indicates that CBD could function as a third-generation TKI, acting against Thr790→Met mutations.

### 4.2. CBD Targets Switch I and Switch II in RAS Isoforms

The RAS family members have the same function and mechanism of action and a high degree of sequence similarity. The first amino acid residues (1–86) are identical in all RAS isoforms except for mutations that occur [34,35,36]. An important pocket within the RAS isoforms (HRAS, KRAS, and NRAS) is the GDP/GTP binding pocket, which comprises two regions of Ras: Switch I (residues 25–40) and Switch II (residues 60–76) [37,38,39]. The RAS isoforms are activated when structural changes occur between Switch I and II, facilitating GDP release and GTP binding [33,35]. As shown here, CBD forms hydrophobic interactions with KRAS with Phe28, Val29, Asp30, Tyr32, and Thr35 (Figure 2C). With these interactions, CBD could possibly bind to the GDP/GTP pocket, subsequently inhibiting the release of GDP and the hydrolysis of GTP. This mechanism alludes to CBD being a novel inhibitor of KRAS.

### 4.3. DFG Motif as a Potential Target of CBD

A DFG motif consists of aspartic acid/phenylalanine/glutamine residues, which regulate the catalysis of a protein kinase and play a role in ATP binding to the kinase [40,41,42]. The highly conserved DFG motif is situated in the C-lobe of the catalytic domain of a kinase and is involved in the magnesium-binding coordination [43]. Type 1 inhibitors bind to the DFG motif in an active conformation and bind to the ATP pocket, preventing substrate protein phosphorylation [44]. In comparison, type 2 kinase inhibitors are ligands that will bind to and occupy the hydrophobic pocket of the kinase, which is adjacent to the ATP binding site [45].

This in silico analysis revealed that CBD would bind to the kinase domain of BRAF through a hydrogen bond formed with the Asp594 residue of the kinase domain (Figure 2A). This residue forms part of the larger C-lobe, which is involved in binding substrate proteins [46] and the DFG motif [40,41,42]. In the active state of BRAF, Asp594 forms a bond with a divalent magnesium ion, stabilizing the phosphate groups of ATP [46]. CBD also occupies the hydrophobic pocket of BRAF, located between Val471, Cys532, Trp531, Thr529, Leu514, and Ala481 (Figure 2A) [37,42]. As this hydrophobic pocket is next to the catalytic loop, potentially with the binding of CBD to the hydrophobic pocket, the activity of the catalytic loop will be impaired [46]. This inhibition will consequently prevent the transfer and phosphorylation of BRAF’s downstream target proteins, indicating the potential use of CBD as either a type 1 or 2 kinase inhibitor.

The present analysis predicted that CBD would interact with MEK1 via hydrophobic interactions with Gly77, Val82, Ala95, Lys97, Leu118, Val127, Ile141, Met143, Leu197, Cys207, Asp208, Phe209, and Val211 (Figure 2D). Important interactions include CBD with Lys97, Met143, Asp208, and Phe209. Notably, Asp208, and Phe209 are part of the DFG motif in MEK1, which functions similarly to the DFG motif of BRAF [47]. The Asp208 residue plays a critical role in the active and inactive state of MEK1, the orientation of this residue determining the active and inactive states of BRAF [48]. Asp208 is the first residue in the MEK1 activation loop, which will bind Mg^2+^ ions that will subsequently be involved in the coordination of the phosphates of ATP [49]. If the DFG motif is in the inactive conformation, the Asp208 residue will face away from the active site; meanwhile, in the active conformation, it will face the active site [44,46,47]. Thus, the interaction of CBD with Asp208 and Phe209 will block switching from the inactive to the active state, resulting in the activation loop impeding the binding site of ERK1 on MEK1 [44,48].

In the inactive conformation of the DFG motif, an allosteric binding site or hydrophobic pocket will be formed next to the DFG motif. This pocket will be separated from the DFG motif by Lys97 and Met143 [50,51,52]. Importantly, Lys97 is part of a conserved region of three amino acids that forms a Lys-Asp-Asp motif, which plays a key role in phosphoryl transfer [48]. This is an essential catalytic residue involved in coupling the ATP phosphates, forming a salt bridge with a Glu114, an occurrence that signals that the kinase is in the active catalytic state [43,44,49]. The binding of CBD to this residue will prevent the formation of the salt bridge, limiting the transfer of a phosphoryl group, so trapping the kinase in an inactive state. Type 1 MEK1 inhibitors have a similar chemical structure to ATP and will compete with it for the catalytic binding site on MEK1 [48,53]. Type 2 inhibitors function in occupying the hydrophobic pocket next to the ATP-binding pocket when the DFG motif is in its inactive state. These inhibitors will form hydrogen bonds with Glu114 and Asp208, forming van der Waals interactions between other residues in this region [44,46,53].

### 4.4. Additional Targets for CBD-Induced Inhibition

The in silico analysis identified additional MAPK-pathway proteins that also interact with CBD; however, theses predicted interactions are slightly weaker than those described above. These CBD-PR interactions include cRAF, NRAS, HRAS, MEK2, ERK1, and ERK2, which form the MAPK signaling cascade.

**Figure 3:**
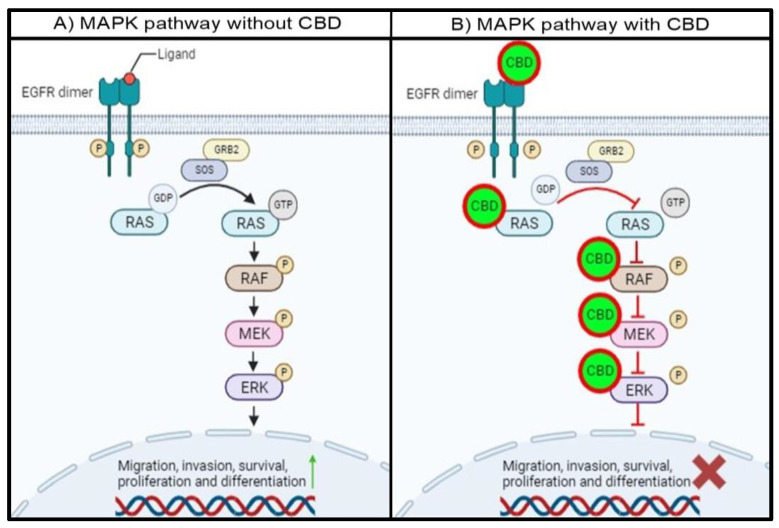
Proposed schematic representation of the MAPK pathway with and without CBD. (**A**) In the absence of CBD, the MAPK pathway remains hyperactivated due to the unregulated activation of EGFR and MAPK pathway proteins. (**B**) In the presence of CBD, the proteins in the EGFR/MAPK pathway are inhibited through the abovementioned mechanisms. This will lead to the cascade-like prevention of target protein activation, resulting in a decrease in the activation and expression of proteins associated with cell migration, survival, and proliferation, among others.

The interaction between CBD and the proteins involved in each step of the MAPK pathway could potentially be used as a novel method to treat CRC. Since CBD interacts with almost all proteins involved in the pathway, it will not only inhibit the activity of a single protein but will also inhibit the activity of multiple MAPK-pathway proteins, as depicted in Figure 3.

In the absence of CBD, the intracellular tyrosine kinase domain of EGFR will be phosphorylated with ligand binding, resulting in the dimerization of EGFR. This phosphorylated residue will provide a docking site for GRB2, which will interact with SOS. This complex will allow GDP-bound RAS to release GDP and hydrolyze GTP to activate RAS, leading to subsequent RAF, MEK1/2, and ERK1/2 activation. The activation of ERK1/2 will lead to the activation and transcription of target proteins and genes associated with migration, invasion, survival, proliferation, and differentiation (Figure 3A).

The proposed mechanism for the interaction between CBD and the MAPK pathway proteins indicates that the entire MAPK pathway will be affected in the presence of CBD. CBD binding to EGFR will prevent the transfer of a phosphoryl group to GDP, which is bound to RAS, leaving RAS inactive. Secondly, the interaction between CBD and RAS will either prevent the release of GDP and hydrolysis of GTP and occupy the ATP binding pocket in the RAS isoforms. This will prevent RAS from phosphorylating RAF, leaving RAF inactive. Thirdly, CBD can also interact with RAF, specifically BRAF, and impair the catalytic activity by binding to the hydrophobic pocket and ATP binding site. This will inhibit the activity of BRAF, and MEK downstream of BRAF will not be activated. Finally, the interaction of CBD with MEK1 will also trap MEK1 in an inactive state by binding to the DFG motif and hydrophobic pocket. Subsequently, the MAPK pathway will be impaired, and the activation and transcription of proteins involved in cell migration, invasion, survival, proliferation, and differentiation will be either decreased or inhibited (Figure 3B). This can also be useful since proteins in the MAPK pathway can often be mutated and become resistant to conventional treatments. With the multi-protein inhibition of the MAPK pathway, treatment resistance can be overcome.

## 5. Conclusions

This in silico study predicts that CBD could play a pivotal role in inhibiting the EGFR and MAPK pathways since almost all the proteins involved in this pathway interact with CBD. The most notable interactions occur between CBD and EGFR, KRAS, BRAF, and MEK1, as reflected by docking scores and being the most critically mutated or dysregulated proteins in colorectal cancer. CBD is proposed to act as an inhibitor of these proteins mainly by binding to the ATP catalytic binding site, which prevents phosphotransfer and the subsequent downstream activation of the substrate proteins. Secondly, CBD can act by binding to the DFG, which is adjacent to the hydrophobic pocket. The catalytic activity of this target protein is inhibited by this mechanism. Since the effect of CBD on these proteins has not yet been investigated, future studies should aim to determine if CBD indeed binds to these predicted target sites in these proteins and if the expected inhibitory effect occurs. Furthermore, in vitro phosphorylation studies on the selected proteins may determine if the phosphorylation of these proteins is affected by CBD treatment. In conclusion, CBD is predicted to interact with multiple role-players in the EGFR and MAPK pathways, potentially inhibiting these pathways and proteins.

## Figures and Tables

**Table 1:** MAPK-pathway proteins identified with their relative Vina docking score and the bonds and interactions involved in CBD binding.

Protein Name	Vina Score	Hydrogen Bond Formed with CBD	Hydrophobic Interactions Formed with CBD
BRAF	−8.9	Asp594	Gly594, Leu505, Phe595, Ile527, Ser467, Leu514, Val471, Lys483, Thr529, Phe583, Ala481, Gln530
EGFR	−8.8	Lys745	Phe723, Leu747, Leu788, Leu858, Asp855, Phe856, Met766, Leu777, Met790
KRAS	−8.5	Lys117, Gly31, Gly13	Lys16, Ser17, Asn116, Ala18, Phe28, Asp30, Val29, Tyr32, Thr35
MEK1	−8.0	None	Phe209, Val211, Leu118, Asp208, Met143, Gly77, Leu197, Val82, Cys207, Ala95, Lys97, Val127, Ile141
ERK2	−7.9	Lys54	Met108, Leu107, Leu156, Ile31, Asn154, Ser153, Tyr36, Asp167, Ile56, Val39, Gln105, Ala52
ERK1	−7.7	Asp184	Gly50, Lys131, Cys183, Gly49, Asp128, Val56, Met125, Ile48, Leu173, Ala52, Gly51, Tyr53, Ser170
NRAS	−7.6	Gly13, Glu31	Ala18, Ile21, Ser17, Asp33, Gly15, Val29, Tyr32, Asp30, Phe28, Lys147, Asp119, Lys117, Val14
cRAF	−7.6	None	Leu476, Trp423, Ser427, Gly426, Ala373, Leu406, Val374, Lys375, Asp486, Val363, Ser357, Gly358, Phe475, Cys424
MEK2	−7.5	Asp212	Ala80, Ser198, Gly81, Gly79, Gly84, Met147, Val86, Gly153, Met150, Ala99, Leu78, Leu201, Asp156, Ser154

## Data Availability

The datasets generated and/or analyzed during the current study and the data not shown here are available from the corresponding author upon reasonable request. As the data files are Excel spreadsheets, they are not included in the Supplementary Materials document. Data required to replicate the study can be found in the Supplementary Materials document.

## Supplementary Materials

The following supporting information can be downloaded at https://www.mdpi.com/article/10.3390/cimb46080506/s1. Table S1: Proteins identified during interaction with CBD and their docking scores and gene names. Table S2: CB-dock results of CBD binding to EGFR. Table S3: CB-dock results of CBD binding to KRAS. Table S4: CB-dock results of CBD binding to HRAS. Table S5: CB-dock results of CBD binding to NRAS. Table S6: CB-dock results of CBD binding to BRAF. Table S7: CB-dock results of CBD binding to cRAF. Table S8: CB-dock results of CBD binding to MEK1. Table S9: CB-dock results of CBD binding to MEK2. Table S10: CB-dock results of CBD binding to ERK1. Table S11: CB-dock results of CBD binding to ERK2.
