# Supplementary material for: A Bioinformatic Analysis Predicts That Cannabidiol Could Function as a Potential Inhibitor of the MAPK Pathway in Colorectal Cancer
Source: Curr Issues Mol Biol. 2024 Aug 5;46(8):8600–10. doi: 10.3390/cimb46080506 (PMC11352951; doi:10.3390/cimb46080506)
Supplement: Supplementary file 1 [file cimb-46-00506-s001.zip › cimb-3092108-supplementary.pdf]

Supplementary data

Table S1: Proteins identified during to interact with CBD as well as their relative docking scores and gene names.

Protein	Gene name	Docking Score
Retinoic acid receptor beta	RARB	10.2
Retinoic acid receptor alpha	RARA	9.9
RAC-alpha serine/threonine-protein kinase	AKT1	9.5
Retinoic acid receptor gamma	RARG	9.4
Retinoic acid receptor RXR-alpha	RXRA	9.3
Tyrosine-protein kinase ABL2	ABL2	9.2
**B-Raf proto-oncogene serine/threonine-protein kinase**	**BRAF1**	**9.1**
Retinoic acid receptor RXR-beta	RXRB	8.9
Cyclin-A2	CCNA2	8.9
Dual specificity protein kinase	CLK1	8.9
Dual specificity protein kinase	CLK2	8.7
Proto-oncogene serine/threonine-protein kinase	PIM1	8.6
Dual specificity protein kinase	CLK3	8.6
Protein tyrosine kinase 2 beta	FAK2	8.6
Mitogen-activated protein kinase 11	*MK11*	*8.6*
Serine/threonine-protein kinase	SGK1	8.6
Dual specificity tyrosine-phosphorylation-regulated kinase 2	DYRK2	8.6
Serine/threonine-protein kinase receptor R3	ACVL1	8.6
**GTPase KRAS**	**RASK**	**8.4**
Mast/stem cell growth factor receptor	KIT	8.4
Eukaryotic translation initiation factor 4E-binding protein 1	4EBP1	8.4
Glycogen synthase kinase-3 beta	GSK3B	8.4
Proto-oncogene tyrosine-protein kinase	FYN	8.3
Mitotic checkpoint serine/threonine-protein kinase	BUB1	8.3
**Dual specificity mitogen-activated protein kinase kinase 1/MEK1**	**MP2K1**	**8.2**
Proto-oncogene tyrosine-protein kinase	SRC	8.1
Proto-oncogene tyrosine-protein kinase receptor	RET	8.1
Mitogen-activated protein kinase kinase kinase 9	*M3K9*	*8.1*
Serine/threonine-protein kinase	PIM2	8
**Epidermal growth factor receptor**	**EGFR**	**7.9**
Death-associated protein kinase 3	DAPK3	7.9
G1/S-specific cyclin-D3 Cell division protein kinase 4	CDK4	7.9
Rho-associated protein kinase 1	ROCK1	7.9
Mitogen-activated protein kinase 9	*MK09*	*7.9*
Mitogen-activated protein kinase 8	*MK08*	*7.9*
**GTPase HRAS**	**RASH**	**7.8**
Cyclin-A2 Cyclin-dependent kinase inhibitor 1B	CDKN1B	7.8
Serine/threonine-protein kinase	CHK1	7.8
Cell division protein kinase 6	CDK6	7.8
MAP kinase-interacting serine/threonine-protein kinase 2	*MKNK2*	*7.8*

TGF-beta receptor type-1	TGFR1	7.8
Proto-oncogene tyrosine-protein kinase	FES	7.8
**Dual specificity mitogen-activated protein kinase kinase 1 MEK1**	**MP2K1**	**7.8**
Activated CDC42 kinase 1	ACK1	7.8
Death-associated protein kinase 1	DAPK1	7.8
Mitogen-activated protein kinase 12	*MK12*	*7.6*
Proto-oncogene tyrosine-protein kinase	LCK	7.5
Cyclin-T1 Cell division protein kinase 9/Cell division protein kinase 9	CDK9	7.5
Mitogen-activated protein kinase 10	*MK10*	*7.4*
Death-associated protein kinase 2	DAPK2	7.4
Mitogen-activated protein kinase kinase kinase 7	*M3K7*	*7.4*
Serine/threonine-protein kinase PAK 7	PAK7	7.1
Serine/threonine-protein kinase PAK 1	PAK1	7.1
Mitogen-activated protein kinase 6	*MK06*	*7.1*
Mitogen-activated protein kinase 1	*MK01*	*7.1*
MAP kinase-activated protein kinase 3	*MAPK3*	*7.1*
Mitogen-activated protein kinase kinase kinase 5	M3K5	7
MAP/microtubule affinity-regulating kinase 3	MARK3	7
Proto-oncogene tyrosine-protein kinase MER	MERTK	7
Mitogen-activated protein kinase 13	*MK13*	*7*

Proteins highlighted in bold have been selected for further docking studies. Proteins highlighted in italics are additional MAPK-like proteins.

Table S2: CB dock results of CBD binding to EGFR.

**Vina score**	**Cavity size**	**Centre**			**Size**		
		**X**	**y**	**z**	**x**	**y**	**z**
-8.8	5079	26	61	-89	27	35	21
-8.1	2914	23	83	-119	21	29	21
-7.5	12875	29	90	-66	35	35	34

Table S3: CB dock results of CBD binding to KRAS:

**Vina score**	**Cavity size**	**Centre**			**Size**		
		X	y	z	x	y	z
-8.5	577	11	28	1	21	30	21
-6.1	122	4	15	12	21	21	21
-5.5	100	14	14	-7	21	21	21

Table S4: CB dock results of CBD binding to HRAS:

Vina Score	Cavity size	Centre			Size		
		X	y	z	x	y	z
-7.3	906	30	-29	0	21	21	21
-6.6	74	22	-40	31	21	21	21
-5.6	84	24	-50	22	21	21	21

Table S5: CB dock results of CBD binding to NRAS

Vina score	Cavity size	Centre			Size		
		X	y	z	x	y	z
-7.6	1013	9	34	20	21	21	21
-5.5	147	-1	20	22	21	21	21
-4.9	62	4	12	4	21	21	21

Table S6: CB dock results of CBD binding to BRAF kinase domain

Vina score	Cavity size	Centre			Size		
		X	y	z	x	y	z
-8.9	3705	-18	-5	-14	29	30	21
-6.8	1567	-33	-17	-48	21	30	21
-5.5	2004	-5	-15	-37	21	28	21

Table S7: CB dock results of CBD binding to cRAF.

Vina Score	Cavity size	Centre			Size		
		X	y	z	x	y	z
-7.6	1425	29	42	32	21	21	21
-6.5	1495	5	16	39	27	21	27
-6.5	1485	19	24	35	21	21	35

Table S8: CB dock results of CBD binding to MEK1.

Vina score	Cavity size	Centre			Size		
		X	y	z	x	y	z
-8	1909	-5	64	31	21	31	21
-6.3	745	5	75	26	21	21	31
-5.7	209	-22	61	10	21	21	21

Table S9: CB dock results of CBD binding to MEK2.

Vina Score	Cavity size	Centre			Size		
		X	y	z	x	y	z
-7.5	1208	24	121	50	21	21	31
-6.3	2711	22	98	42	32	21	34
-5.9	2835	39	113	51	21	21	27

Table S10: CB dock results of CBD binding to ERK1.

Vina Score	Cavity size	Centre			Size		
		X	y	z	x	y	z
-7.7	4382	25	-2	18	21	31	34
-6.7	2601	6	-2	25	28	21	35
-5.4	1295	6	-21	40	30	21	35

Table S11: CB dock results of CBD binding to ERK2.

Vina Score	Cavity size	Centre			Size		
		X	y	z	x	y	z
-7.9	1223	4	11	48	21	21	21
-5.9	1485	-13	-6	31	21	21	21
-5	246	7	-5	48	21	21	21
